# Phenotypic and Molecular Characterization of Nonfermenting Gram-Negative Bacilli Causing Peritonitis in Peritoneal Dialysis Patients

**DOI:** 10.3390/pathogens11020218

**Published:** 2022-02-08

**Authors:** Ana Cláudia Moro Lima dos Santos, Aydir Cecília Marinho Monteiro, Thaís Alves Barbosa, Danilo Flávio Moraes Riboli, Carlos Henrique Camargo, Adriano Martison Ferreira, Alessandro Lia Mondelli, Augusto Cezar Montelli, Rodrigo Tavanelli Hernandes, Maria de Lourdes Ribeiro de Souza da Cunha, Pasqual Barretti

**Affiliations:** 1 Departamento de Ciências Químicas e Biológicas, Instituto de Biociências, Universidade Estadual Paulista Júlio de Mesquita Filho (UNESP), Botucatu 18618-689, SP, Brazil; anna.moro@hotmail.com (A.C.M.L.d.S.); aydir@terra.com.br (A.C.M.M.); thaalvesb@hotmail.com (T.A.B.); daniloflaviomr@yahoo.com.br (D.F.M.R.); rt.hernandes@unesp.br (R.T.H.); 2 Centro de Bacteriologia, Instituto Adolfo Lutz, São Paulo 01246-902, SP, Brazil; btcarlos@gmail.com; 3 Hospital das Clínicas da Faculdade de Medicina de Botucatu, Botucatu 18607-741, SP, Brazil; adrianomartison@hotmail.com; 4 Departamento de Clínica Médica, Faculdade de Medicina, Universidade Estadual Paulista Júlio de Mesquita Filho (UNESP), Botucatu 18618-970, SP, Brazil; alessandro.mondelli@unesp.br (A.L.M.); acmontelli@uol.com.br (A.C.M.); pbarretti@uol.com.br (P.B.)

**Keywords:** peritoneal dialysis, peritonitis, biofilm, antimicrobial resistance, *Pseudomonas*, *Acinetobacter*, nonfermenting Gram-negative bacilli, clonal profile, virulence factors

## Abstract

(1) Background: Peritonitis due to nonfermenting Gram-negative bacilli (NF-GNB) is a dramatic complication of peritoneal dialysis (PD) with bad outcomes. Previous studies of PD-related peritonitis due to *Pseudomonas* species have shown a low-resolution rate, without a high resistance rate to antipseudomonal antibiotics. This suggests that bacterial virulence factors can act and influence peritonitis evolution. This study aimed to describe the microbiological characteristics of NF-GNB causing PD-related peritonitis and analyze their influence on the outcome. (2) Methods: We analyze the 48 isolates from NF-GNB peritonitis, which were stored in our culture collection regarding bacterial resistance, biofilm, and other virulence factors’ production, and clonal profile. Additionally, we collected data on treatment and outcomes from patients’ clinical registers. (3) Results: The etiologies were species of *Pseudomonas* (50%), *Acinetobacter* (36%), and other NF-GNB (14%). There was a high (75%) proportion of biofilm producer lineages. The in vitro susceptibility rate of *Pseudomonas* spp. to amikacin, ciprofloxacin, and ceftazidime was significantly greater than that of *Acinetobacter* spp. and other species; however, there was a similar low-resolution rate (<45%) among the episodes attributable to them. *Pseudomonas* species have a polyclonal profile, while we found a clone of five multiresistant *Acinetobacter baumannii* over an 8-year interval (2000–2008), which suggest an origin from the healthcare environment. (4) Conclusions: We are not able to identify any predictor of outcome, but it is possible that biofilm and others virulence factors can act in concert and contribute to the bad outcome.

## 1. Introduction

Despite the substantial decrease in peritonitis rates over recent decades [1,2], it remains a major complication for chronic kidney disease patients undergoing peritoneal dialysis (PD) [2,3]. Our group reported that peritonitis is the most common cause of PD discontinuation in Latin America [4]. Davenport [3] showed similar results in London’s dialysis centers, as well as de Moraes et al., in the largest Latin American cohort of PD patients [5]. In addition, peritonitis has been associated with long-term cardiovascular deaths [6].

Usually, PD-related peritonitis progresses to resolution [7]; however, its outcome is strongly influenced by the causal microorganism. Episodes caused by *Staphylococcus aureus* [8] and Gram-negative bacilli (GNB), especially nonfermenting GNB (NF-GNB), present a more severe clinical course and a high non-resolution rate [9,10].

The reasons for the unfavorable outcome of NF-GNB peritonitis are not fully known. Despite the indisputable role of bacterial resistance, this does not seem to be the only property influencing the outcome. The two largest previous series on PD-related peritonitis caused by *Pseudomonas* species showed poor outcomes. The first included 191 Australian patients and reported high rates of catheter removal (44%) and hemodialysis transfer (35%) [11]. The second, which included 153 episodes in Hong Kong, reported a resolution rate of 42.4%, in addition to a decrease in the bacterial resistance rate to ceftazidime and gentamicin over time [12].

NF-GNB produces several virulence factors, particularly *Pseudomonas* species, that can produce exoenzyme S, an inhibitor of protein synthesis; hemolytic phospholipase C, which is related to the destruction of cell membranes; exotoxin A, a promoter of tissue destruction and macrophage response inhibition; alkaline protease, a causer of tissue damage and inactivation of IgG; elastase, an immunoglobulin degradation factor; rhamnolipids, which are associated with bacterial adhesion; and alginate, which actively participates in the production of the biofilm, a polysaccharide matrix that surrounds the microorganisms, forming colonies; along with other virulence factors. Biofilm formation represents a protective growth mode that allows microorganisms to survive in hostile environments. Clinically, biofilms are responsible for many persistent and chronic infections because of their inherent resistance to antimicrobial agents, due to the difficulty antimicrobials find in penetrating this protective matrix [13,14,15,16,17,18].

A previous study by our group was not able to identify which clinical factors, treatments employed, or microbiological factors of the NF-GNB causing peritonitis on peritoneal dialysis were related to the outcomes of this infection; only exit-site infection (ESI) was identified as an independent predictor of non-resolution of these infections [19]. Then, the pathogenic profile of NF-GNB may explain, at least partially, the bad outcomes of PD-related peritonitis. We aimed to describe microbiological characteristics of NF-GNB species causing peritonitis in a single university dialysis center, as antimicrobial susceptibility, adding data on virulence factors and clonal profile of these microorganisms. To our knowledge, no previous publication has been designed with this objective.

## 2. Results

### 2.1. Sampling and Identification

From 1997 to 2015, there were 70 episodes of NF-GNB peritonitis. We retrieved 48 isolates from these episodes from our culture collection for microbiological testing. The microorganisms isolated and patient information are listed in Table 1.

The epidemiology of these isolates in relation to the studied period was varied; we only observed a concentration of episodes caused by *P. aeruginosa* in the years 1998–1999 (seven cases) and in the year 2006 (seven cases). In relation to the 48 episodes, 50.0% (24 cases) were caused by *Pseudomonas* spp., 37.5% (18 cases) were caused by *Acinetobacter* spp., 6.2% (three cases) were caused by *Achromobacter* spp., 4.2% (two cases) were caused by *Burkholderia* spp*.,* and one case (2.1%) by *Stenotrophomonas maltophilia* (date reproduced from Dos Santos et al.) [19].

With respect to the features of the patients, 56.2% were male, and 56.2% were below 50 years of age (Table 1). In the 48 episodes of peritonitis due to NF-GNB, 24 patients were treated by CAPD, and 24 were treated by APD.

### 2.2. In Vitro Susceptibility

*Pseudomonas* species had a higher susceptibility rate than *Acinetobacter* species to all antimicrobials, except for imipenem. Additionally, *Pseudomonas* had a higher susceptibility rate than *Achromobacter* species to amikacin, ciprofloxacin, and cefepime. *B. cepacia* and *S. maltophilia* were tested only for ceftazidime and were susceptible (Table 2).

In the period studied, we observed that the isolates causing peritonitis in the years 1997 to 1998 were sensitive to all antibiotics tested in this study, with only one *P. aeruginosa* strain having intermediate sensitivity to ciprofloxacin. From 1999 to 2010, the resistance profile was variable, and we observed 13 isolates (35.1%) with resistance profiles to more than three classes of antibiotics, namely eight *A. baumannii*, two *P. aeruginosa*, one *A. haemolyticus*, and one *B. gladioli*. The isolates from the years 2013 to 2015 showed sensibility to all the antibiotics tested. Therefore, we did not observe significant changes in the resistance pattern of these isolates during the entire period studied.

### 2.3. Biofilm Production

Of the 48 samples, 35 (75%) were biofilm producers, including 18 strong, 7 medium, and 10 weak biofilm producers. The biofilm producers consisted of 22 *Pseudomonas* isolates, 11 *Acinetobacter* isolates, 1 *Achromobacter* isolate, and 1 *S. maltophilia* isolate. Two *Pseudomonas* isolates, seven *Acinetobacter* isolates, two *Achromobacter* isolates, *B.* *cepacia*, and the *B. gladioli* isolate were not biofilm producers. Results reproduced from Dos Santos et al. [19].

### 2.4. Virulence Profile

All isolates from *P. aeruginosa* were positive for the alginate D, hemolytic C, non-hemolytic phospholipase C, exotoxin A, alkaline protease, and elastase genes, in contrast with 21 (87.5%) for rhamnolipids and 14 (58.3%) for exoenzyme S.

### 2.5. Clonal Profile

This analysis was performed for the species *P. aeruginosa* and *A. baumannii* due to their higher prevalence. Only 17 *P. aeruginosa* isolates and 9 *A. baumannii* isolates were included, because at the time of the pulsed-field gel electrophoresis (PFGE) analysis, it was not possible to recover the other isolates.

**Figure 1:**
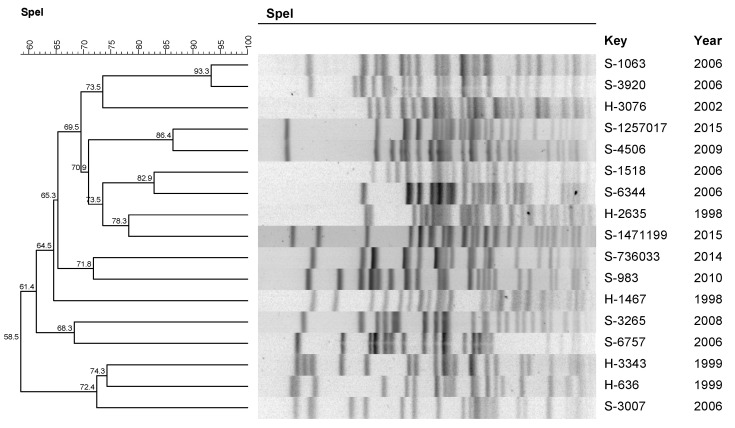
Dendrogram generated by the Dice/UPGMA analysis (Bionumerics, AppliedMaths) of the PFGE *Spe*I profiles of *P. aeruginosa* isolated from peritonitis in patients treated with peritoneal dialysis.

The *P. aeruginosa* profile (Figure 1) was polyclonal, although there was a high degree of similarity in three clusters with two isolates in each cluster (A, B, and C). It is also noted that cluster B revealed a similarity of 86.4% between the sample S-4506 isolated in 2009 and the sample S-1257017 isolated in 2015 (Table 3). The microbiological characteristics of the isolates that presented 80% or more similarity are described in Table 3.

**Figure 2:**
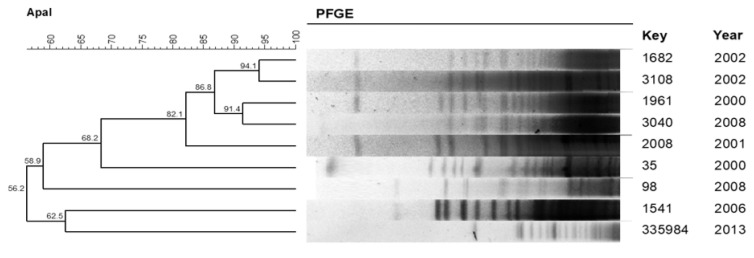
Dendrogram generated by the Dice/UPGMA analysis (Bionumerics, AppliedMaths) of the PFGE *Apa*I profiles of the *A. baumannii* isolated from peritonitis in patients treated with peritoneal dialysis.

PFGE revealed an important *A. baumannii* clone with the cluster of five isolated samples from August 2000 to June 2008 and with 82.1% similarity (Figure 2). The microbiological characteristics of the *A. baumannii* isolates that presented 80% or more similarity are described in Table 4, where a multi-resistant profile of these isolates is evidenced.

### 2.6. Peritonitis Outcomes

Patients initial (empirical) treatment was based on ISPD guidelines for empirical (ini-tial treatment) [7,20,21], which recommend a coverage for both Gram-positive cocci and Gram-negative bacilli. When the results of peritoneal effluent culture and in vitro susceptibility tests are available, the treatment is adjusted. For the episodes caused by *Pseudomonas* spp. and *Stenotrophomonas*, an association of two susceptible antipseudomonal antibiotics is recommended, [7]; for the others, the adjustment is made based on “in vitro susceptibility”.

In our sample, for 24 episodes caused by *Pseudomonas* spp., the adjusted treatment was: two antipseudomonal drugs in 23 cases (97.8%) (amikacin plus ciprofloxacin, in 13 and amikacin plus ceftazidime in 10 episodes) and no adjustment in one, in which immediate catheter removal occurred. We observed an episode caused by *S. maltophilia*, in which ceftazidime plus ciprofloxacin was used for adjustment. For the 18 *Acinetobacter* spp. Episodes, the adjustment occurred in 100.0% of the cases and was based on monotherapy with imipenem in nine cases, amikacin in eight cases, and ceftazidime in one case. The duration of antibiotic therapy was at least 21 days [7].

Of the 48 episodes, we found 13 resolutions (27.1%); 4 relapses (8.3%); 17 refractory peritonitis episodes (35.4%); 12 catheter removals on the fifth day of treatment (25.0%) that occurred in episodes caused by seven isolates of *P. aeruginosa*, one isolate of *P. putida*, one isolate of *A. denitrificans*, one isolate of *S. maltophilia*, and two isolates of *A. baumannii*; and two peritonitis-related deaths (4.2%), caused by one isolate of *A. baumannii* and one isolate of *A. ursingii*.

The resolution rate was 20.8% for the 24 infections caused by *Pseudomonas*, 38.8% for the 18 caused by *Acinetobacter*, and 16.6% in peritonitis caused by other NF-GNB species (*p* = 0.35).

### 2.7. Predictors of Peritonitis Outcome—Logistic Regression Analysis

Only biofilm production was associated with the dependent variable of non-resolution of peritonitis (*p* = 0.11). Therefore, we are not able to apply a multiple regression model.

## 3. Discussion

Peritonitis in patients treated for PD has decreased in recent years [4,22,23] but remains a major cause of technique failure, also contributing to increased morbidity and mortality.

In this center and in the period studied, the rate of peritonitis caused by Gram-negative bacilli was 26.7% of all episodes. A similar frequency was found by Prasad et al. [24], and 9.5% of these episodes were caused by BGNNF.

As previously published by our group [19], the results of the present study confirmed the predominance of *P. aeruginosa* species as etiologic agents of BGNNF peritonitis [10] in patients treated for PD. *Pseudomonas* peritonitis is usually severe and is often associated with catheter infection. Retrospective studies have shown that peritonitis by this microorganism is associated with higher frequencies of hospitalization, high rates of catheter removal, and permanent hemodialysis transfer [11,25].

The study of virulence factors of *P. aeruginosa* revealed the presence of genes encoding pathogenicity factors in 100% of isolates, except for rhamnolipids and exoenzyme, present in 87.5% and 58.3% of isolates, respectively. When comparing these results with other studies, we found the same percentage present of the genes studied in samples from patients with cystic fibrosis [26]. Another study also conducted in Brazil by Gonçalves et al. [27] showed that virulence genes were present in a high percentage of strains (88.0%) from infections from various sources of infections and hospital origin. On the other hand, the percentages of frequencies of these genes are lower in studies conducted with samples from urinary tract infections, and another with samples from water and soil [18,28]. These findings show that virulence genes, although present in most strains [29], differ according to the origin of the isolate.

Our results confirm a high production of biofilm among NF-GNB, in particular, *P. aeruginosa*. In turn, a high resistance rate was observed among NF-GNB strains, except for *Pseudomonas* species, which were highly susceptible (about 80%) to the majority of antibiotics, except for ciprofloxacin. These findings suggest that bad outcomes observed in this study (resolution rate < 40%) and in the two largest previous series [11,12], which reported PD-related peritonitis caused by *Pseudomonas* species, are a consequence of its aggressive virulence profile beyond antibiotic resistance.

However, biofilm production is a potential determinant of an unfavorable antimicrobial response. The routine antibiotics prescription is based on the MIC of the drug for planktonic cells, which are more sensitive to antimicrobials than their counterparts wrapped in a biofilm [16,30]. We found a high proportion of biofilm-producing isolates (75%), which may have hindered the therapeutic response [31].

We were not able to identify a single virulence factor as a predictor of the outcome, and this analysis was hampered by the finding of their production in almost all isolates. Although individual analysis of the pathogenic factors did not show an association with the outcome, this does not rule out the possibility that pathogenic action of these may contribute to a bad outcome.

There was an important proportion of episodes due to species of *Acinetobacter*, and their resistance to several antimicrobials was confirmed in this series, except for imipenem, and this becomes a major therapeutic challenge [32]. Clonal profile revealed a cluster of *A. baumannii* with five samples, isolated within an interval of 8 years with a multi-resistance profile to antimicrobials. These bacteria are commonly found in hospital environment infections [33], and the presence of a clone for a long interval suggests that peritonitis caused by this agent can have a hospital source. *Acinetobacter* species cause PD-related peritonitis, which is difficult to treat, with a high rate of catheter removal and technique failure [34,35]. In contrast, *P. aeruginosa* species presented a polyclonal profile suggesting different origins, probably from the patient’s microbiota or their environment.

We identified microorganisms such as the *Achromobacter* species, *B. cepacia*, and *B. gladioli*, which have been rarely described as PD-related peritonitis etiologies. The precise identification of NF-GNB represents a challenge for conventional microbiology, due to the phenotypic similarity and taxonomic complexity of these agents. Phenotypic tests based on morphology and biochemical characteristics often provide erroneous identifications. In our study, this was minimized with the identification of isolates by means of matrix-assisted laser desorption ionization time-of-flight (MALDI-TOF), a technique that identifies microorganisms based on their protein profile.

Our study has several limitations, in addition to the small sample size. The financial resources for this study came from a grant from the by São Paulo Research Foundation (FAPESP), which ended in 2015. As the microbiological assays that were performed are not used in daily routine practice, it was not possible to proceed with sample follow-up after 2015. During this study, we performed the laboratory techniques at different times, and the last performed was the clonal typing of the isolates (PFGE). Initially, we recovered the 48 isolates that were stored at −80 °C, and after performing the first techniques (identification, in vitro susceptibility, biofilm production and virulence profile of *P. aeruginosa*), we refrozen these strains again. When we went to recover them again to perform the clonal typing of the isolates, for reasons unknown to us, seven strains of *P. aeruginosa* and two strains of *A. baumannii* did not grow again. Because we have had a few isolates included in the study, we left the results of the other techniques for all isolates (48 strains), without excluding the strains not included in the PFGE technique. However, to our knowledge, it was the first study that addressed the virulence factors intrinsic to these germs and their potential role in outcomes. In addition, it provided novel information about pathogens rarely identified as the genus *Achromobacter*, which suggests the benefits of the use of new techniques such as MALDI-TOF for bacteria identification in peritonitis.

## 4. Materials and Methods

We studied all isolates of NF-GNB from PD-related peritonitis that occurred between 1997 and 2015 that are stored in our culture collection. Routinely, in our dialysis center, after bacterial identification and susceptibility testing, strains causing peritonitis are stored frozen at –80 °C. In addition, we recorded from patients’ clinical register data on treatment and episode outcome.

The institutional research ethics committee approved this study and exempted it of any specific written informed consent.

### 4.1. Sampling and Identification

The stored samples were re-isolated on MacConkey agar plates (Oxoid) and reidentified. The isolates were Gram-stained to confirm purity and to determine morphology and specific color. Afterwards, they were identified by conventional biochemical methods [20]. In addition, they were identified by mass spectrometry using MALDI-TOF technology VITEK^®^ MS (bioMérieux, Marcy-l’Étoile, France) [36].

### 4.2. In Vitro Susceptibility

We tested the susceptibility to amikacin, ciprofloxacin, cefepime, imipenem, and ceftazidime by means of the minimal inhibitory concentration (MIC) obtained by the E-test (bioMérieux, Marcy-l’Étoile, France), made on the basis of the Clinical Laboratory Standards Institute (CLSI) based on the 2019 Clinical Laboratory Standards Institute (CLSI) breakpoints [37].

### 4.3. Biofilm Production

The samples were grown in TSB at 37 °C for 18 h. To assess the bacterial ability to adhere to abiotic surfaces, we used polystyrene plates with 96 wells, each of which added 200 µL of TSB and 10 µL of the bacterial suspension (approximately 10^8^ CFU/mL). In addition, we inoculated a well only with culture medium to be used as a reading standard (blank). The inoculated plates were incubated at 37 °C for 48 h and then washed with Phosphate-Buffered Saline 4 times to remove non-adherent bacteria. Adherents were fixed with formalin (2%), and after 20 min, they were removed, and the preparations were washed 4 times with water. Further to this, the preparations were stained with a crystal violet solution (1%) for 20 min, after which, they were washed 3 times with water to remove excess dye. After the drying period, the dye was solubilized with methanol for 10 min, and the optical density (OD) measured at 540 nm was determined [38]. Then, we classified the biofilm production into one of the four categories: non-producer (OD of strain < OD of blank); weak producer (OD of strain 2 times OD of blank), medium producer (DO of strain = 2–4 times OD of blank); and strong producer (OD of strain ≥ 4 times OD of blank).

### 4.4. Virulence Profile of Pseudomonas aeruginosa

We used the polymerase chain reaction (PCR), in which the samples were evaluated for the presence of the genes encoding alginate (*algD*), elastase (*lasB*), hemolytic phospholipase C (*plcH*), non-hemolytic phospholipase C (*plcN*), exoenzyme S (*exoS*), exotoxin A (*toxA*), alkaline protease (*lasB*), and rhamnolipid (*rhlAB*). The protocol, for each reaction and primer, was performed as described by Lanotte et al. [26]. The amplifications were performed using the following cycles: initial denaturation at 94 °C for 3 min, followed by 30 cycles: denaturation at 94 °C for 30 s, annealing at 58 °C for 1 min, and extension at 72 °C for 1.5 min, followed by a final extension at 72 °C for 5 min. The amplified products were visualized after electrophoresis on a 2% agarose gel stained with Syber safe using UV light to visualize the bands.

### 4.5. Clonal Profile

The *P. aeruginosa* isolates and *A. baumannii* isolates obtained from patients on PD were submitted to chromosomal DNA restriction analysis with 30U of the enzyme *Spe*I and 50U of *Apa*I (New England Biolabs, Ipswich, EUA), respectively. We followed the protocol by Durmaz et al. [39] for *A. baumannii* and the PulseNet protocol (*Escherichia coli* O157: H7, *Escherichia coli* non O157 (STEC), serotypes of *Salmonella*, *Shigella sonnei,* and *Shigella flexneri*) for *P. aeruginosa*. The DNA profiles generated by PFGE were inserted into the BioNumerics software, version 7.6 (Applied Maths, Sint-Martens-Latem, Belgium) for analysis. Dendrograms were built for each species, with optimization and tolerance parameters set at 1.0 and 1.5, respectively. Two or more isolates with a similarity coefficient of 80% or higher were chosen for the definition of clusters.

### 4.6. Clinical-Microbiological Associations

Antibiotic treatment and microbiological characteristics were studied regarding their association with the peritonitis outcome. Using the International Society for Peritoneal Dialysis (ISPD) criteria [7], the outcomes were: resolution, refractoriness, catheter removal before 5 days from the treatment start, relapse, and peritonitis-related death.

### 4.7. Statistical Analysis

We compared frequencies using the chi-square test or Fisher’s exact test and used binary logistic regression with backward stepwise to determine predictors of outcome, which were classified in two mutually results: resolution or nonresolution (refractoriness, relapse, and peritonitis-related death). To select the variables to multivariate model, we used a *p* value > 0.20 as the elimination criterion from univariate analysis. A *p* value < 0.05 was considered significant.

## 5. Conclusions

Our results confirm that NF-GNB PD-related peritonitis is a serious infection with a very low-resolution rate. Bacterial resistance, particularly among episodes caused by *Pseudomonas* species, is not enough to explain the bad outcome. No individual virulence factor was associated with the outcome, which does not rule out the possibility that they will act in concert, impairing the response to antimicrobial therapy. The high prevalence of multi-resistant *Acinetobacter* species causing PD-related peritonitis, and with a clonal profile suggesting hospital origin, raises alarm about the care for the prevention and management of these infections.

## Figures and Tables

**Table 1:** Etiology, year, and patient data from 48 episodes of peritonitis caused by non-fermenting Gram-negative peritonitis bacilli in patients undergoing peritoneal dialysis at the University Hospital of the Botucatu Medical School, São Paulo, Brazil.

			Patient Data		
Sample Number	Species	Year of Isolation	Age (Years)	Gender	Dialysis *
H 1196	*Acinetobacter ursingii*	1997	46	M	CAPD
H 829	*Pseudomonas aeruginosa*	1998	14	M	CAPD
H 1467	*Pseudomonas aeruginosa*	1998	24	M	CAPD
H 2635	*Pseudomonas aeruginosa*	1998	54	F	CAPD
H 167	*Pseudomonas aeruginosa*	1999	51	F	CAPD
H 636	*Pseudomonas aeruginosa*	1999	9	M	CAPD
H 2624	*Pseudomonas aeruginosa*	1999	73	F	CAPD
H 3343	*Pseudomonas aeruginosa*	1999	6	M	CAPD
H 3379	*Acinetobacter haemolyticus*	1999	29	F	CAPD
H 35	*Acinetobacter baumannii*	2000	65	F	DPA
H 1961	*Acinetobacter baumannii*	2000	40	M	CAPD
H 2008	*Acinetobacter baumannii*	2001	69	M	CAPD
H 1682	*Acinetobacter baumannii*	2002	81	F	DPA
H 2337	*Burkholderia gladioli*	2002	74	M	CAPD
H 3076	*Pseudomonas aeruginosa*	2002	43	M	CAPD
H 3108	*Acinetobacter baumannii*	2002	66	M	CAPD
S 22813	*Acinetobacter haemolyticus*	2003	52	M	DPA
S 23517	*Pseudomonas aeruginosa*	2003	21	F	DPA
S 22270	*Acinetobacter ursingii*	2004	49	F	CAPD
S 20514	*Pseudomonas pútida*	2005	44	M	CAPD
S 1045	*Achromobacter denitrificans*	2006	56	F	DPA
S 1063	*Pseudomonas aeruginosa*	2006	42	F	DPA
S 1518	*Pseudomonas aeruginosa*	2006	62	M	CAPD
S 1541	*Acinetobacter baumannii*	2006	54	M	DPA
S 3007	*Pseudomonas aeruginosa*	2006	35	M	CAPD
S 3920	*Pseudomonas aeruginosa*	2006	65	M	CAPD
S 6344	*Pseudomonas aeruginosa*	2006	2	F	DPA
S 6430	*Pseudomonas aeruginosa*	2006	46	M	DPA
S 6757	*Pseudomonas aeruginosa*	2006	33	F	DPA
S 7122	*Acinetobacter haemolyticus*	2006	35	F	DPA
S 2797	*Achromobacter denitrificans*	2007	24	M	DPA
S 3620	*Acinetobacter baumannii*	2007	61	M	CAPD
S 4411	*Pseudomonas putida*	2007	54	M	CAPD
S 5215	*Burkholderia cepacia*	2007	53	M	DPA
S 98	*Acinetobacter baumannii*	2008	91	F	CAPD
S 3040	*Acinetobacter baumannii*	2008	32	F	DPA
S 3265	*Pseudomonas aeruginosa*	2008	78	F	DPA
S 4661	*Achromobacter denitrificans*	2008	22	F	DPA
s 4845	*Acinetobacter haemolyticus*	2008	26	M	CAPD
S 500	*Acinetobacter haemolyticus*	2009	27	M	DPA
S 4506	*Pseudomonas aeruginosa*	2009	36	M	CAPD
S 56765	*Stenotrophomonas maltophilia*	2009	42	M	DPA
S 983	*Pseudomonas aeruginosa*	2010	54	M	DPA
S 1489	*Acinetobacter baumannii*	2010	46	F	DPA
S 335984	*Acinetobacter baumannii*	2013	66	F	DPA
S 736033	*Pseudomonas aeruginosa*	2014	15	F	DPA
S 1257017	*Pseudomonas aeruginosa*	2015	30	F	DPA
S 1471199	*Pseudomonas aeruginosa*	2015	64	M	DPA

* Automatic peritoneal dialysis (APD) and continuous ambulatory peritoneal dialysis (CAPD).

**Table 2:** Non-fermenting Gram-negative bacilli-causing peritoneal dialysis-related peritonitis episodes and their in vitro susceptibility rates by minimal inhibitory concentration, reproduced from Dos Santos et al. [19].

	*Pseudomonas* spp. (*n* = 24)	*Acinetobacter* spp. (*n* = 18)	*Achromobacter* spp. (*n* = 3)	*B. gladioli* (*n* = 1)	*B. cepacia* (*n* = 1)	*Stenotrophomonas* spp. (*n* = 1)	NF-GNB (*n* = 48)
	Susceptibility *n* (%)	Susceptibility *n* (%)	Susceptibility *n* (%)	Susceptibility *n* (%)	Susceptibility *n* (%)	Susceptibility *n* (%)	Susceptibility *n* (%)
Amikacin	20 (83.3) ^1,2^	7 (38.9)	1 (33.3)	1 (100)	-	-	29 (60.4)
Ciprofloxacin	17 (70.1) ^1^	7 (38.9)	2 (66.7)	0 (0.0)	-	-	26 (54.1)
Ceftazidime	21 (87.3) ^1^	8 (44.4)	3 (100)	0 (0.0)	1 (100.0)	1 (100.0)	34 (70.8)
Cefepime	20 (83.3) ^1,2^	7 (38.9)	1 (33.3)	0 (0.0)	-	-	27 (56.2)
Imipenem	20 (83.3)	16 (88.9)	3 (100)	0 (0.0)	-	-	39 (81.2)

^1^ = *p* < 0.05 vs. *Acinetobacter* spp., ^2^ = *p* < 0.05 vs. *Achromobacter* spp.

**Table 3:** Characterization of virulence and antimicrobial profile of *P. aeruginosa*.

Cluster	Similarity	Sample	Year	Antimicrobial Profile *		Biofilm Production	Virulence Genes **
				Sensibility	Resistance		
A	93.3%	S-1063	2006	IPM	AMI, CAZ, COM and CIP	Strong producer	*algD, exoS, plcH, plcN, toxA, aprA, lasB, e rhlAB*
		S-3920	2006	-	AMI, CAZ, CPM, CIP and IPM	Strong producer	
B	86.4%	S-4506	2009	AMI, CAZ, CPM, CIP and IPM	-	Strong producer	*algD, exoS, plcH, plcN, toxA, aprA, lasB, e rhlAB*
		S-1257017	2015	AMI, CAZ, CPM, CIP and IPM	-	Moderate producer	
C	82.9%	S-1518	2006	AMI, CAZ and CPM	CIP and IPM	Moderate producer	*algD, plcH, plcN, toxA, aprA, lasB, e rhlAB*
		S-6344	2006	CAZ, CPM and IPM	CIP and AMI	Moderate producer	

* AMI: amikacin, CAZ: ceftazidime, CPM: cefepime, CIP: ciprofloxacin e IPM: imipenem. ** *algD*: alginate, *exoS*: exoenzyme S, *plcH*: hemolytic phospholipase C, *plcN*: non-hemolytic phospholipase C, *toxA*: exotoxin A, *aprA*: alkaline protease, *lasB*: elastase e *rhlAB*: rhamnolipid.

**Table 4:** Characterization of the antimicrobial profile and biofilm production of *A. baumannii* isolates grouped in a cluster.

Cluster	Similarity	Sample	Year	Antimicrobial Profile *		Biofilm Production
				Sensibility	Resistance	
A	82.1%	S-1961	2000	IPM	AMI, CAZ, CPM, and CIP	Weak producer
		S-2008	2001	IPM	AMI, CAZ, CPM, and CIP	Weak producer
		S-1682	2002	IPM	AMI, CAZ, CPM, and CIP	No producer
		S-3108	2002	IPM and CPM	AMI, CAZ, and CIP	Weak producer
		S-3040	2008	IPM and CPM	AMI, CAZ, and CIP	No producer

* AMI: amikacin, CAZ: ceftazidime, CPM: cefepime, CIP: ciprofloxacin e IPM: imipenem.

## Data Availability

Correspondence and requests for materials should be addressed to M.d.L.R.d.S.d.C.
